# Bioinformatic screening and detection of allergen cross‐reactive IgE‐binding epitopes

**DOI:** 10.1002/mnfr.201600676

**Published:** 2017-03-27

**Authors:** Scott McClain

**Affiliations:** ^1^ Syngenta Crop Protection LLC Durham NC USA

**Keywords:** Allergen, Bioinformatics, Cross‐reactivity, Epitope, FASTA, Homology

## Abstract

Protein allergens can be related by cross‐reactivity. Allergens that share relevant sequence can cross‐react, those lacking sufficient similarity in their IgE antibody‐binding epitopes do not cross‐react. Cross‐reactivity is based on shared epitopes that is based on shared sequence and higher level structure (charge and shape). Epitopes are important in predicting cross‐reactivity potential and may provide the potential to establish criteria that identify homology among allergens. Selected allergen's IgE‐binding epitope sequences were used to determine how the FASTA algorithm could be used to identify a threshold of significance. A statistical measure (expectation value, *E*‐value) was used to identify a threshold specific to identifying cross‐reactivity potential. Peanut Ara h 1 and Ara h 2, shrimp tropomyosin Pen a 1, and birch tree pollen allergen, Bet v 1 were sources of known epitopes. Each epitope or set of epitopes was inserted into random amino acid sequence to create hypothetical proteins used as queries to an allergen database. Alignments with allergens were noted for the ability to match the epitope's source allergen as well as any cross‐reactive or other homologous allergens. A FASTA expectation value range (1 × 10^−5^–1 × 10^−6^) was identified that could act as a threshold to help identify cross‐reactivity potential.

**Abbreviations**
*E*‐value: expectation value
NCBI: National Center for Biotechnology Information

## 1 Introduction

Cross‐reactivity has generally been studied in the context of food safety. The goal has been to understand how sensitization to one particular food may allow for cross‐reactivity to homologous allergens in other foods. Cross‐reactivity begins with sensitization to one allergen. Exposure to another highly similar protein that shares enough of the IgE‐binding epitope structure/sequence can also sensitize and/or elicit an allergic response. This shared sequence and shared reactivity is termed, cross‐reactivity. In some cases, a sensitizer may not cause allergy symptoms and the cross‐reactive allergen is the eliciting antigen, but not sensitizing to the patient. This example illustrates the case where the eliciting, cross‐reactive allergen is an incomplete allergen. In contrast, the allergen that can both sensitize and elicit a reaction is considered a “true” or complete allergen. Nevertheless, it is the shared sequence homology that is of interest in determining the potential to elicit a clinically relevant allergy response, that is, an allergy event associated with symptomology such as wheezing, urticaria, and oral allergy syndrome, as examples.

Protein allergens have the potential to cross‐react if two or more allergens share amino acid sequence to a degree that there is shared IgE antibody binding. IgE binding is ascribed to the IgE‐binding regions, or epitopes, of the allergen. Shared IgE binding across multiple proteins is based on the premise that there is high degree of homology within the epitope(s) to maintain IgE binding. Thus, cross‐reactivity results from the Fc epsilon‐RI (FcεRI) receptor binding by the IgE–allergen complex from either allergen 1. Together, this complex is the basis for stimulating mast cells and basophils to release mediators, such as histamine.

IgE‐binding epitopes can be of a sequential, uninterrupted amino acid string or as a discontinuous distribution throughout the larger allergen sequence. A sequential epitope tends to result in a minimum length of sequence that can bind IgE 2, 3, and in some food allergen based cases retain IgE‐binding capacity after surviving gastric enzyme reduction of the intact protein 4. Sequential epitopes may be part of larger epitopes, but likely have the same physical constraints in binding IgE whether or not they are isolated as peptides or within the intact protein 5. Still, sequential epitopes acting to bind IgE as part of the larger allergen structure would of course be impacted by the constraints of structure and charge imparted by the rest of the sequence. Discontinuous epitopes (e.g., conformational or nonsequential) are defined by an allergen remaining intact and the maintenance of proper folding (secondary and tertiary sequence conformation) to bring distributed residues within range of one another to allow IgE binding.

The structural family of allergens with the most numerous sequences is the birch tree pollen allergens (Bet v 1). This allergen group is well recognized for birch tree respiratory sensitivity, which can be due to sensitivity to one or more of the many isoforms produced by *Betula pendula* (verrucosa) species 6. However, the allergen belongs to a broader group of structurally related proteins in several species, some of which can induce allergy based on shared homology. This allergen group is a good example of epitope homology where patients with birch pollen hay fever can also experience clinical symptoms not from the original sensitizing allergen, Bet v 1, but are instead reacting to a Bet v 1 homologue in a food. One allergen that shares homology with Bet v 1 is Mal d 1; the pathogen resistance associated protein in apples that can cause oral allergy syndrome 7. The Bet v 1 sequence appears to be the “parental” source of the shared epitopes, as all of the Mal d 1 epitopes (both B and T‐cell) are contained within Bet v 1. Clinically, the focus is on the elicitation response and this is shown by Bet v 1 being able to inhibit the B‐cell epitopes through IgE binding by Mal d 1, but with Mal d 1 being unable to fully inhibit Bet v 1 IgE binding 7. It should be noted that the foods themselves are not exclusively dependent on the Bet v 1 as a sensitizer. The foods themselves can also prompt naïve or original reactivity to the allergens in those foods directly 8. It should be recognized that other proteins in birch pollen and foods with the Bet v 1 homologues may also sensitize and elicit allergy of their own accord.

Bioinformatics has the capacity to statistically determine the probability of taxonomic relatedness at the protein level 9, 10. As Pearson (2000) notes, “…, with biological sequences (as opposed to fair coins), the assumptions underlying the statistical model may not be met. When the assumptions fail, the highest scoring unrelated sequence may have an expectation value (*E*‐value) that is much too low (e.g., *E* < 10^−3^] or much too high [*E* > 100]” 11. This sets the context for using FASTA as a tool, which needs to be vetted for its use in specific cases with appropriate context for the groups of proteins being evaluated. Bioinformatics has been extended herein in its application for assessing whether similarity can describe the possibility of cross‐reactivity between protein allergens. The shared percent identity in amino acids remains a traditional way to describe how alike two proteins are in their sequence. Although noted for the imperfect nature of using identity (i.e., a percentage of shared, exact amino acid matches across a total amino acid length) to “find” potential cross‐reactivity among sequences 5, an identity threshold has found its way into regulatory guidance 12, 13. Thus, the metric of a minimum 35% shared identity, plus a minimum of 80 amino acid overlap length, has become criteria to establish significant shared sequence between an unknown or novel protein allergen and a known allergen. In the regulatory framework from 2001 (FAO/WHO; evaluation of allergenicity of genetically modified foods), the intent was to set a tiered approach whereby the first step would be that if an alignment between an allergen and a novel protein exceeded 35% and 80 amino acid overlap, then a second step, serum screening, would be employed to confirm the existence or absence of cross reactivity. However, as was recognized at the time, there was no qualified, complete list of known allergens 14, which could be systematically explored for similarity thresholds. Together with the fact that very few epitopes for allergens were known at the time, the 35% over 80 amino acids represented a conservative approach to setting a tiered assessment that hinged on serology‐based confirmation of allergenicity, but lacked a detailed exploration of allergens and the way in which cross‐reactivity can be assessed bioinformatically.

It is well understood that bioinformatics and alignment algorithms base their probability assessments of homology on extrapolating to higher order protein structure from sequential sequence similarity (i.e., identical and similar residues). In the case of the FASTA algorithm, the intent is to identify local alignments between sequences 11 to find the portions of two proteins that may describe their core areas of shared sequence. This local alignment feature of FASTA is consistent with how epitopes tend to be localized as small portions within the larger, intact protein sequence. In the current study, verified IgE‐binding epitopes of key allergens were used to determine a minimum alignment threshold to detect homologous sequences. In using only epitope sequence information, the focus was on testing the capacity of minimum, but immunologically relevant sequence to act as a bioinformatic screen for other homologous or cross‐reactive allergens. Known, sequential and discontinuous epitopes were used to model the localized positioning of the epitopes within a larger protein sequence. Hypothetical query proteins were constructed of random amino acid sequence that was modified to include known allergen epitopes; in effect, doping random sequence with known, biologically relevant allergen epitopes. Each hypothetical protein was then compared to a database of known allergens to determine whether FASTA alignments could discern homology based on only epitopes. In using epitopes, the goal was to model the use of bioinformatics for establishing threshold criteria based on biological similarity among distinct allergen groups.

## 2 Methods

Random amino acid sequence was used to construct hypothetical protein sequence(s) that were of the same length as known allergens. Random sequence was used to fill in between the portions of known allergen epitopes. This random “filler” sequence was derived from random, alternative open reading frame sequence, as derived and translated from an original gene, human alpha‐amylase; the filler sequence otherwise had no similarity to the parental gene or any known gene, including allergens. The actual primary reading frame was ignored, and a reverse reading frame was translated and prepared using the BLAST program, GETORF3 routine 15; this amino acid sequence was then randomized (Supporting Information Fig. 1). Portions of each filler sequence were repetitively used to construct hypothetical proteins of the proper length for each allergen from which epitopes were derived (discussed below).

### 2.1 Hypothetical sequence construction based on allergen epitopes

Each hypothetical sequence was prepared by spacing the known epitopes and placing them into their same locations, relative to the N‐terminus of the native allergen. Allergen sequences were based on their identification, as listed in the Food Allergy Research and Resource www.allergenonline.org database 16, 17.

**Figure 1:**
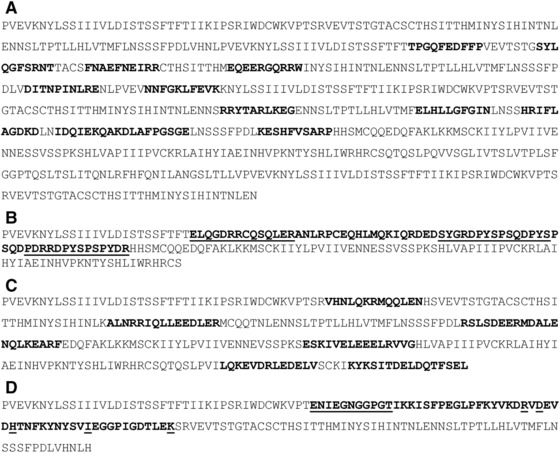
(A) A random selection of amino acids loaded with peanut Ara h 1 epitopes, numbers 10–22: total length = 626. Epitopes are identified by bold and highlighted lettering. (B) A random selection of amino acids was loaded with the epitopes of peanut Ara h 2 (AH2‐1, AH2‐3a, and AH2‐3c); total length = 172 aa. A contiguous epitope region covering 66 amino acids is identified by bold and highlighted lettering; underlined epitopes are, in order from N‐ to C‐terminal end, AH2‐1, AH2‐3a, and AH2‐3c. (C) A random selection of amino acids was loaded with shrimp Pen a 1 epitopes: total length = 284. Epitopes are identified by bold and highlighted lettering. (D) A random selection of amino acids was loaded with the region of sequence from Bet v 1 containing the discontinuous epitope residues; total length = 160 aa. Epitope region is identified by bold and highlighted lettering; underlined letters are the Bet v 1 epitope residues.

To create the hypothetical sequence for Ara h 1, the IgE‐binding 10‐mer peptides that were identified by the Bannon laboratory 18, 19 were placed into random sequence to create a 626 amino acid sequence to be used for allergen database comparisons (Fig. 1A). The length was based on the Ara h 1 protein (GI: 1168391) with epitope locations mapped into the hypothetical sequence according to their native location in Ara h 1 18.

A second peanut‐based allergen sequence was constructed based on the recent discovery that nonhomologous proteins may have cross‐reactivity 20. A hypothetical sequence was constructed using synthetic epitopes known to cross‐react with peanut allergens. The epitopes AH2‐1, AH2‐3a, and AH2‐3c from Bublin et al. 20 were loaded into random sequence to make a 172 amino acid length sequence (Fig. 1B) based on the Ara h 2 protein (GI: 26245447). A supplementary 172 amino acid hypothetical sequence was also prepared, but a contiguous 69 amino acid section covering the AH2‐1, AH2‐2, and AH2‐3a AH2‐3b and AH2‐3c epitopes 20 was inserted into random sequence.

The epitopes of the Pen a 1 tropomyosin allergen from the brown shrimp species *Penaeus aztecus* 21 were used to prepare hypothetical sequence as with the other allergens; note, the genus is now listed as *Farfantepenaeus*. Epitope regions were inserted based on concatenating the individual, overlapping epitopes listed in the work by Reese, et al., Fig. 1A–E 21. The length is based on the Pen a 1 protein (GI: 73532979) and was used to create a hypothetical protein sequence of 284 amino acids (Fig. 1C).

The epitope 22 of the European White birch (*B. pendula*) pollen allergen, Bet v 1, was prepared similar to the others. The single epitope is discontinuous along the length of the allergen. The length is based on the average length of several listed isoforms of the Bet v 1 protein (example given by GI: 1542865) with random sequence used to create a 160 amino acid hypothetical protein (Fig. 1D). In addition to only using the epitope residues, a separate hypothetical sequence was constructed using the entire region of the Bet v 1 protein over which the epitopes are dispersed. This “epitope region” is 56 amino acids in length and two separate sequences were constructed for comparing with the allergen database. It should be noted that in using only one epitope this is not necessarily a representation of the multiple, nonoverlapping eptitopes required by an allergen for cross‐linking of the FcεRI receptor 1.

### 2.2 FASTA comparisons

Each hypothetical sequence was compared to a protein allergen sequence database (FARRP, 2015). The database consisted of 1,897 sequences representing clinically confirmed, as well as putative allergens. The comparison was performed using the FASTA algorithm, version 3.4t11 10. Parameter settings for FASTA were as follows: BLOSUM 50 matrix, gap penalty = 12, gap extension penalty = 2, *Z* = 2,000, and *z* = 1. A minimum 30% shared identity and a sequence overlap length of 40 amino acids were used as display limits for the output shown in tables; an upper threshold of *E* = value of 10 was also used for display of alignments. This combination of alignment display limiting was set below the Codex Alimentarius (2009) guideline values of 35% and 80 amino acids to insure alignments would be displayed above and below the Codex threshold values.

## 3 Results

The alignments produced between each epitope‐containing sequence and allergens were evaluated to identify homologous and possible cross‐reactive allergens. Four sequences were used; peanut Ara h 1 and Ara h 2, shrimp tropomyosin Pen a 1 and the birch tree pollen allergen, Bet v 1. They have varying levels of taxonomic conservation at the epitope level and across the entire sequence that were expected to affect alignment metrics 23, 24. Tropomyosin, for example, has well‐recognized conservation across many species that was expected to allow identification of all known tropomyosin allergens. In order to perform the analysis in this study, each allergen had representative, cross‐reactive epitopes inserted into random, hypothetical sequence. FASTA was used to compare the hypothetical “full‐length” protein to the allergen database in order to observe the primary alignment to any database sequences below the expectation cut‐off of 10 (*E*‐value = 10). Relevant alignments were judged by whether or not aligned database sequences matched the allergen (or group of allergens) from which the epitopes were derived, and by the *E*‐value at which non‐homologous alignments were observed. It should be noted that two or more significantly aligning sequences does not imply known cross‐reactivity in every case. Although highly cross‐reactive groups of allergens have been examined, serology work to support cross‐reactivity to confirm cross‐reactivity has not been performed for many members of the respective allergen homologues discussed herein.

In the first exercise, the 10‐mer peptides that were identified by the Bannon laboratory 18, 19 were examined for their impact on alignments between allergens and a hypothetical 626 amino acid sequence. FASTA analysis showed that the overlap with the parental Ara h1 protein was between 305 and 328 amino acids in length, with the best alignment producing an *E*‐value of 5.3 × 10^−39^ (Table 1). Only Ara h 1 and two beta‐conglycinin proteins from soybean showed alignments, both vicilin‐like 7S globulins 25, 26; the least significant alignment was 2.1 × 10^−11^. Alignments indicated little variance among these homologous proteins, as there were no unexpected allergens from other species identified. The epitopes supported very specific identification of homology even though each epitope was only ten amino acids (except for the case of the overlapping region). The epitopes in total represented 19% of the overall hypothetical sequence.

**Table 1:** Summary of hypothetical sequence comparisons to an allergen database

	Most significant alignment		Least significant alignment	
Allergens used for hypothetical sequence	*E*‐value	Species, allergen	*E*‐value	Species, allergen
Ara h 1	5.3 × 10^−39^	*Arachis hypogea*, Ara h 1	2.1 × 10^−11^	*Glycine max*, β‐conglycinin
Bet v 1, residues only	6.8 × 10^−3^	*Betula pendula*, Bet v 1	8.0 × 10^−1^	*Vigna radiata*, Vig r 1.0101 (PRP 10 protein)
Bet v 1, 56 aa epitope region	1.1 × 10^−22^	*Betula pendula*, Bet v 1	3.0 × 10^−3^	*Vigna radiata*, Vig r 6.0101
Pen a 1	3.4 × 10^−27^	*Metapenaeus ensis*, Hom a 1.0102 homologue	1.5 × 10^−9^	*Tyrophagus putrescentiae*, Tyr p 10
Ara h 2	5.1 × 10^−17^	*Arachis hypogea*, Ara h 2	5.1 × 10^−10^	*Arachis hypogea*, Ara h 2.01 allergen

Significance is based on the summary FASTA statistic, *E*‐value; a small value is more significant. Least significant alignment displayed in this table is that which is *E*‐value ≤ 9.9 × 10^−1^.

The Pen a 1 allergen is the tropomyosin protein from shrimp with a length of 284 amino acids (FARRP 2015), and it represents a highly conserved protein with less variability across species than Ara h 1. The percentage of epitope residues relative to the overall sequence length was 32%, the most of any of the hypothetical proteins. FASTA results showed a total of 72 alignments, with all of these far below an *E*‐value of 0.1 (Table 1). The most significant alignment was nearly identical in its *E*‐value to several other tropomyosins from related species (35 alignments had *E*‐values between 1.3 × 10^−26^ and 8.1 × 10^−22^), indicating close homology within this structurally related group. The source organism of the Pen a 1 sequence is the shrimp species, *Farfantepenaeus aztecus*, and it displayed the second most significant alignment (*E*‐value 1.5 × 10^−26^).

The analysis of Bet v 1 was focused on exploring whether a discontinuous epitope 22 would retain the capacity to represent the protein in general; that is, could the epitope alone flag Bet v 1 (and homologues) when inserted into random sequence. Sixteen epitope residues (Fig. 1D) were considered as a challenge in the use of FASTA due to the lower concentration of contiguous residues as well as the lower total number of residues relative to the random sequence length (10%). The hypothesis was that algorithms such as FASTA may be limited in identifying either the parental sequence or homologues with so few epitope residues scattered throughout the sequence. As shown in Table 1, the Bet v 1 allergen was clearly observed (as the most significant alignment) as were numerous other members of this large, cross‐reactive allergen family (Tables 2 and 3). In all, there were 106 alignments (alignment data not shown) with only two alignments producing an *E*‐value greater than the cut‐off of 0.1 used for Table 1. An *E*‐value range of 0.001–0.01 is recommended as an upper threshold below which alignments likely start to have significance 11. Most of the other species with Bet v 1 homologues were represented, including genus’ *Castanea*, *Corylus*, *Malus*, *Quercus*, and *Carpinus* and the range in *E*‐values was between 6.8 × 10^−3^ and 8.0 × 10^−1^; a very narrow range.

**Table 2:** All alignments comparing the hypothetical protein containing the Bet v 1 epitope region with the allergen database

Database match description	GI number	Species	% Identity	Overlap	*E*‐value
Pollen allergen Betv1, isoform At8	4006928	*Betula pendula*	58.8	114	1.10E‐22
Pollen allergen Betv1, isoform At37	4006953	*Betula pendula*	58.8	114	1.50E‐22
Bet Vi Jap1	12583681	*Betula platyphylla*	57.9	114	1.70E‐22
Isoallergen Bet V 1 B1	4590392	*Betula pendula*	57.9	114	1.70E‐22
Pollen allergen Bet V 1	1542861	*Betula pendula*	57.9	114	1.70E‐22
Bet Vi Jap3	12583685	*Betula platyphylla*	57.9	114	2.00E‐22
Pollen allergen , Betv1	4376216	*Betula pendula*	55.7	115	2.00E‐22
Chain A, birch pollen allergen Bet V 1 mutant N28t, K32q, E45s, P108g	11514622	*Betula pendula*	57.9	114	2.80E‐22
Pollen allergen Bet V 1	1542869	*Betula pendula*	87.9	66	3.30E‐22
Bet V 1 D	452732	*Betula pendula*	57	114	3.30E‐22
Bet V 1 L	452744	*Betula pendula*	57	114	3.30E‐22
Major pollen allergen Bet V 1‐	1168706	*Betula pendula*	57	114	3.30E‐22
Pollen allergen Bet V 1	1542867	*Betula pendula*	58.8	114	3.30E‐22
Pollen allergen Betv1	2564220	*Betula pendula*	57	114	3.30E‐22
Pollen allergen Bet V 1	1542865	*Betula pendula*	57.9	114	3.90E‐22
Chain A, birch pollen allergen Bet V 1	159162097	*Betula pendula*	56.5	115	4.50E‐22
Bet Vi Jap2	12583683	*Betula platyphylla*	57.9	114	4.60E‐22
Pollen allergen Betv1, isoform At14	4006947	*Betula pendula*	86.4	66	5.20E‐22
Pollen allergen Betv1, isoform At7	4006967	*Betula pendula*	57	114	5.30E‐22
Pollen allergen Bet V 1	1542873	*Betula pendula*	55.7	115	5.30E‐22
Bet V 1 ‐ like	17938	*Betula pendula*	86.4	66	6.30E‐22
chain A, crystal structure of a dimeric variant of Bet V 1	565807648	*Betula pendula*	86.4	66	6.30E‐22
Chain A, crystal structure of a variant of the major birch pollen allergen Bet V 1	560188693	*Betula pendula*	86.4	66	6.30E‐22
Variant of Bet V chain A of the crystal structure	560188694	*Betula pendula*	86.4	66	6.30E‐22
Chain B, crystal structure of a dimeric variant of Bet V 1	560188692	*Betula pendula*	86.4	66	6.30E‐22
Isoallergen Bet V 1 B2	4590394	*Betula pendula*	86.4	66	6.30E‐22
Major allergen Bet V 1	1321720	*Betula pendula*	86.4	66	6.30E‐22
Pollen allergen Betv1	2564224	*Betula pendula*	86.4	66	6.30E‐22
Pollen allergen Betv1, isoform At5	4006965	*Betula pendula*	86.4	66	6.30E‐22
Pollen allergen Bet V 1	1542863	*Betula pendula*	84.8	66	7.40E‐22
Pollen allergen Betv1, isoform At42	4006955	*Betula pendula*	84.8	66	7.40E‐22
Pollen allergen Betv1	2564228	*Betula pendula*	56.1	114	7.40E‐22
Pollen allergen Betv1, isoform At87	4006963	*Betula pendula*	84.8	66	8.40E‐22
Pollen allergen Betv1, isoform At45	4006957	*Betula pendula*	54.8	115	8.70E‐22
Chain A, birch pollen allergen Bet V 1 mutant E45s	38492423	*Betula pendula*	86.4	66	1.00E‐21
Pollen allergen Bet V 1	1542871	*Betula pendula*	84.8	66	1.00E‐21
Pollen allergen Betv1	2564222	*Betula pendula*	84.8	66	1.00E‐21
Pollen allergen Betv1, isoform At50	4006959	*Betula pendula*	84.8	66	1.00E‐21
Pollen allergen Betv1, isoform At10	4006945	*Betula pendula*	84.8	66	1.20E‐21
Bet V 1 F	452736	*Betula pendula*	83.3	66	1.90E‐21
Bet V 1 J	452740	*Betula pendula*	83.3	66	1.90E‐21
Pollen allergen , Betv1	4376222	*Betula pendula*	83.3	66	1.90E‐21
Major allergen Bet V 1	2414158	*Betula pendula*	81.8	66	3.10E‐21
Isoallergen Bet V 1 B3	4590396	*Betula pendula*	53	115	3.10E‐21
Major allergen Bet V 1	1321716	*Betula pendula*	81.8	66	3.70E‐21
Pollen allergen , Betv1	4376220	*Betula pendula*	80.3	66	6.90E‐21
Bet V 1 E	452734	*Betula pendula*	81.8	66	7.00E‐21
Major birch pollen allergen Bet V 1.010, chain A of the crystal structure	550544347	*Betula pendula*	83.3	66	1.10E‐20
Major allergen Bet V 1	1321728	*Betula pendula*	81.8	66	2.10E‐20
Pollen allergen Betv1, isoform At59	4006961	*Betula pendula*	51.8	114	1.50E‐19
1‐Sc1	534910	*Betula pendula*	72.2	72	2.80E‐19
Bet V 1 C	452730	*Betula pendula*	74.2	66	7.30E‐19
Bet V 1 K	452742	*Betula pendula*	74.2	66	7.30E‐19
Bet V 1b	450885	*Betula pendula*	74.2	66	7.30E‐19
Major pollen allergen Bet V 1‐M/	1168710	*Betula pendula*	74.2	66	7.30E‐19
Major allergen Bet V 1	1321724	*Betula pendula*	74.2	66	7.30E‐19
Major allergen Bet V 1	1321718	*Betula pendula*	75.8	66	8.60E‐19
Pollen allergen , Betv1	4376219	*Betula pendula*	75.8	66	8.60E‐19
Pollen allergen , Betv1	4376221	*Betula pendula*	75.8	66	8.60E‐19
Major allergen Bet V 1	1321722	*Betula pendula*	74.2	66	1.00E‐18
1 Sc2	534900	*Betula pendula*	70.8	72	1.40E‐18
1 Sc‐3	534898	*Betula pendula*	77	61	1.90E‐18
Major allergen Cor A 1	1321731	*Corylus avellana*	80.4	56	4.30E‐18
Major allergen Bet V 1	1321726	*Betula pendula*	78.7	61	4.30E‐18
Aln G I	261407	*Alnus glutinosa*	77	61	6.90E‐18
Major allergen Bet V 1	1321714	*Betula pendula*	77	61	6.90E‐18
Pollen allergen Car B 1	1545897	*Carpinus betulus*	78.6	56	1.80E‐17
Pollen allergen Car B 1	1545895	*Carpinus betulus*	76.8	56	1.70E‐16
Car B I, partial	402747	*Carpinus betulus*	76.8	56	2.00E‐16
Major allergen variant Cor A 1.0402	11762102	*Corylus avellana*	68.9	61	3.30E‐16
Major allergen variant Cor A 1.0403	11762104	*Corylus avellana*	68.9	61	3.30E‐16
Major allergen Cor A 1.0401	5726304	*Corylus avellana*	68.9	61	3.90E‐16
Major allergen variant Cor A 1.0404	11762106	*Corylus avellana*	68.9	61	5.30E‐16
Car B I, partial	402743	*Carpinus betulus*	73.2	56	1.40E‐15
Pollen allergen Car B 1	1545877	*Carpinus betulus*	73.2	56	2.60E‐15
Car B I	402745	*Carpinus betulus*	73.2	56	3.10E‐15
Pollen allergen	300872535	*Ostrya carpinifolia*	73.2	56	3.10E‐15
Pollen allergen Car B 1	1545879	*Carpinus betulus*	73.2	56	3.10E‐15
Pollen allergen Car B 1	1545881	*Carpinus betulus*	73.2	56	3.10E‐15
Pollen allergen Car B 1	1545875	*Carpinus betulus*	73.2	56	3.10E‐15
Pollen allergen Car B 1 isoform	167472843	*Carpinus betulus*	73.2	56	3.10E‐15
Pollen allergen Car B 1 isoform	167472841	*Carpinus betulus*	73.2	56	3.10E‐15
Pollen allergen Car B 1 isoform	167472839	*Carpinus betulus*	73.2	56	3.10E‐15
Pollen allergen Car B 1 isoform	167472837	*Carpinus betulus*	73.2	56	3.10E‐15
Pollen allergen Car B 1	1545893	*Carpinus betulus*	66.1	62	3.10E‐15
Pollen allergen Car B 1 isoform	167472845	*Carpinus betulus*	71.4	56	3.60E‐15
Pollen allergen Car B 1	1545889	*Carpinus betulus*	65.6	61	6.90E‐15
Major allergen	22690	*Corylus avellana*	73.2	56	8.10E‐15
Major allergen	22686	*Corylus avellana*	54.5	88	2.10E‐14
Major allergen	22688	*Corylus avellana*	71.4	56	2.50E‐14
Major allergen	22684	*Corylus avellana*	71.4	56	2.50E‐14
Major allergen Cor A 1	1321733	*Corylus avellana*	62.3	61	1.70E‐13
Ypr10	16555781	*Castanea sativa*	63.9	61	2.40E‐13
Fag S 1 pollen allergen	212291472	*Fagus sylvatica*	58.1	62	1.60E‐12
Fag S 1 pollen allergen	212291474	*Fagus sylvatica*	59.7	62	2.20E‐12
Cherry‐allergen PRUA1	1513216	*Prunus avium*	35.5	121	3.60E‐12
Fag S 1 pollen allergen	212291470	*Fagus sylvatica*	58.1	62	5.00E‐12
Mal D 1	747852	*Malus domestica*	43.3	90	1.10E‐11
Major allergen Mal D 1	4768879	*Malus x domestica*	43.3	90	1.10E‐11
Major allergen Mal D 1	4590382	*Malus x domestica*	43.3	90	1.10E‐11
Major allergen Mal D 1	4590376	*Malus x domestica*	43.3	90	1.10E‐11
Major allergen Mal D 1	4590378	*Malus x domestica*	43.3	90	1.10E‐11
Major allergen Mal D 1	4590364	*Malus x domestica*	43.3	90	1.10E‐11
Ribonuclease‐like PR‐10c	15418742	*Malus domestica*	43.3	90	1.10E‐11
Major allergen Pyr C 1	14423877	*Pyrus communis*	44.8	87	1.50E‐11
Major cherry allergen Pru Av 1 mutant E45w, chain A,	159162378	*Prunus avium*	34.7	121	1.50E‐11
Group 2 Car B 1 = isoallergenic variant	1008580	*Carpinus betulus*	79.5	39	1.80E‐11
Major allergen	886683	*Malus x domestica*	43.3	90	1.80E‐11
Major allergen Mal D 1	4590388	*Malus x domestica*	35.9	117	1.80E‐11
18 Kd winter accumulating protein C	54311119	*Morus bombycis*	38.9	113	2.10E‐11
Major allergen Pru P 1	82492265	*Prunus persica*	34.7	121	2.10E‐11
Ap15	862307	*Malus x domestica*	42.5	87	2.90E‐11
Major allergen Mal D 1	2443824	*Malus x domestica*	42.5	87	2.90E‐11
Major allergen D 1	21685277	*Malus x domestica*	42.5	87	2.90E‐11
Major allergen Mal D 1	4590366	*Malus x domestica*	42.5	87	2.90E‐11
Major allergen Mal D 1	4590380	*Malus x domestica*	42.2	90	2.90E‐11
Major allergen Mal D 1	4590368	*Malus x domestica*	42.5	87	3.40E‐11
Pollen allergen Que A 1 isoform	167472849	*Quercus alba*	52.1	73	4.00E‐11
18 Kda winter accumulating protein	610664572	*Morus alba var. atropurpurea*	38.1	113	4.00E‐11
Pollen allergen Que A 1 isoform	167472851	*Quercus alba*	57.4	61	5.50E‐11
Major allergen Mal D 1	1313966	*Malus x domestica*	48.1	77	5.50E‐11
Major allergen Mal D 1	27922941	*Malus x domestica*	48.1	77	5.50E‐11
Putative allergen Pru Du 1.01	190613871	*Prunus dulcis x Prunus persica*	33.9	121	5.50E‐11
Major cherry allergen Pru Av 1.0201	44409451	*Prunus avium*	35.9	117	6.50E‐11
Ribonuclease‐like PR‐10b	15418738	*Malus domestica*	48.1	77	7.60E‐11
Ribonuclease‐like PR‐10a	15418744	*Malus domestica*	41.4	87	7.60E‐11
18 Kd winter accumulating protein A	54311115	*Morus bombycis*	55.4	56	1.00E‐10
Major allergen Mal D 1	1313968	*Malus x domestica*	55.7	61	1.50E‐10
Major allergen Mal D1	1313972	*Malus x domestica*	55.7	61	1.50E‐10
Major cherry allergen Pru Av 1.0202	44409474	*Prunus avium*	49.3	67	1.50E‐10
Major Cherry allergen Pru Av 1.0203	44409496	*Prunus avium*	49.3	67	1.50E‐10
Group 1 Car B 1 = isoallergenic variant	1008578	*Carpinus betulus* = hornbeams, pollen, Peptide Recomb (80 aa)	74.4	39	2.80E‐10
Group 1 Car B 1 = isoallergenic variant	1008579	*Carpinus betulus*	74.4	39	2.80E‐10
Major allergen Mal D1	1313970	*Malus* × *domestica*	54.1	61	3.20E‐10
Putative allergen Rub I 1	110180525	*Rubus idaeus*	41.6	77	6.50E‐10
Major strawberry allergen Fra A 1‐C	90185688	*Fragaria x ananassa*	45.8	72	9.90E‐10
Fra A 1‐A allergen	88082485	*Fragaria x ananassa*	45.8	72	1.00E‐09
Major strawberry allergen Fra A 1‐B	90185682	*Fragaria* × *ananassa*	45.8	72	1.00E‐09
Major strawberry allergen Fra A 1‐D	90185684	*Fragaria* × *ananassa*	45.8	72	1.00E‐09
Pollen allergen Que A 1 isoform	167472847	*Quercus alba*	53.2	62	1.20E‐09
Major allergen protein homolog	2677826	*Prunus armeniaca*	51.8	56	1.60E‐09
Pathogenesis‐related protein	18744	*Glycine max*	47.8	69	1.90E‐09
PR10 protein	565380238	*Solanum lycopersicum*	34.4	93	1.90E‐09
TSI‐1 protein	2887310	*Solanum lycopersicum*	34.4	93	2.00E‐09
Chain B, crystal structure of the strawberry pathogenesis‐related 1	550544407	*Fragaria x ananassa*	44.4	72	2.20E‐09
Major strawberry allergen Fra A 1‐E	90185692	*Fragaria x ananassa*	44.4	72	2.20E‐09
Cas S 1 pollen allergen	212291464	*Castanea sativa*	58.5	53	2.60E‐09
PR10 Protein	565380268	*Solanum lycopersicum*	35.4	96	3.60E‐09
Cas S 1 pollen allergen	212291468	*Castanea sativa*	56.6	53	4.20E‐09
Cas S 1 pollen allergen	212291466	*Castanea sativa*	56.6	53	5.80E‐09
Pathogenesis‐related protein 10	60418924	*Vigna radiata*	43.8	73	9.20E‐09
Bet V 1 related allergen	281552898	*Actinidia deliciosa*	42.5	80	2.90E‐08
Ara H 8 allergen	37499626	*Arachis hypogaea*	42.5	73	8.80E‐08
Ara H 8 allergen isoform 3	169786740	*Arachis hypogaea*	42.5	73	8.80E‐08
Bet V 1 related allergen	281552896	*Actinidia chinensis*	34.7	95	3.20E‐07
Pathogenesis‐related protein 10	110676574	*Arachis hypogaea*	40.3	72	4.40E‐07
Ara H 8 allergen isoform	145904610	*Arachis hypogaea*	33.3	81	9.60E‐07
PRP‐like protein	302379159	*Daucus carota*	34.6	78	5.70E‐06
PRP‐like protein	302379157	*Daucus carota*	34.6	78	6.60E‐06
PRP‐like protein	302379147	*Daucus carota*	33.3	78	1.10E‐05
PRP‐like protein	302379149	*Daucus carota*	33.3	78	1.10E‐05
PRP‐like protein	302379155	*Daucus carota*	32.1	78	2.80E‐05
Pathogenesis‐related protein‐like protein 1	19912791	*Daucus carota*	33.3	78	2.80E‐05
Major allergen Api G	14423646	*Apium graveolens*	32.9	76	2.90E‐05
PRP‐like protein	302379151	*Daucus carota*	32.1	78	5.30E‐05
PRP‐like protein	302379153	*Daucus carota*	32.1	78	5.30E‐05
Cytokinin‐specific binding protein	4190976	*Vigna radiata*	31.3	96	3.00E‐03
Per A 4 allergen	60678787	*Periplaneta americana*	39.6	48	6.30E+00
Vacuolar serine protease	12005497	*Penicillium oxalicum*	35.8	53	7.50E+00

Rows indicated by shaded cells in the *E*‐value column have a percent identity <35 and/or an overlap <80.

**Table 3:** The best (lowest *E*‐value) 15 alignments comparing the hypothetical protein containing the Bet v 1 epitopes only with the allergen database

Database match description	GI number	Species	% Identity	Overlap	*E*‐value
1‐Sc1	534910	*Betula pendula*	30.4	79	6.80E‐03
Bet v 1 c	452730	*Betula pendula*	33.3	63	8.90E‐03
Bet v 1 k	452742	*Betula pendula*	33.3	63	8.90E‐03
Bet v 1b	450885	*Betula pendula*	33.3	63	8.90E‐03
Major pollen allergen Bet v 1‐M/	1168710	*Betula pendula*	33.3	63	8.90E‐03
Major allergen Bet v 1	1321724	*Betula pendula*	33.3	63	8.90E‐03
Major allergen Bet v 1	1321718	*Betula pendula*	33.3	63	8.90E‐03
Pollen allergen Betv1, isoform at59	4006961	*Betula pendula*	33.3	63	8.90E‐03
Pollen allergen, Betv1	4376221	*Betula pendula*	33.3	63	8.90E‐03
Pollen allergen, Betv1	4376219	*Betula pendula*	33.3	63	8.90E‐03
Car b I, partial	402747	*Carpinus betulus*	32.1	56	1.20E‐02
Pollen allergen Car b 1	1545895	*Carpinus betulus*	30.2	63	1.20E‐02
Pollen allergen Betv1, isoform at7	4006967	*Betula pendula*	33.9	56	1.50E‐02
Major allergen Bet v 1	1321722	*Betula pendula*	31.7	63	1.50E‐02
Bet v 1 e	452734	*Betula pendula*	35.7	56	1.80E‐02

Rows indicated by shaded cells in the *E*‐value column have a percent identity <35 and an overlap <80.

In an attempt to determine how Bet v 1 epitope impacted the FASTA *E*‐value range, the entire native protein sequence encompassing the Bet v1 epitope residues (56 amino acids; Fig. 1D) was also loaded into random sequence, similar in construction as the other hypothetical proteins, and compared against the allergen database. The expectation was that homologues of Bet v 1 would be more easily distinguished due to the much greater proportion of the allergen represented in the hypothetical sequence. The sequence consisted of 35% Bet v1 sequence and produced a total alignment count that was greater (169) than using just the epitope residues. As before, there were only two alignments above an *E*‐value of 0.1. The main difference across all the alignments was that most of the isoforms of Bet v 1 represented in allergen database are observed (63) and many more representative isoforms from *Carpinus* and *Caucus*, for example, populate the alignment list (Table 2). The main impact on the alignment metrics was a much more significant *E*‐value maximum (10^−22^ vs. 10^−3^) when the larger 56 amino acid region was used. This is an expectation with FASTA when a large localized portion of the sequence is an exact match 10. The cut‐off between homologues and nonhomologous sequences was also more in line with an established threshold for evaluating allergens with an *E*‐value of 3.9 × 10^−7^ 27 when the larger 56 amino acid Bet v 1 section was used (Table 2).

One last example of epitope detection was examined based on the recent discovery that nonhomologous proteins may have cross‐reactivity 20. The synthetic peptides AH2‐1, AH2‐3a, and AH2‐3c aligned with Ara h 2 and these peptides showed cross‐reactivity by IgE‐binding inhibition 20. These same three peptides were loaded into random sequence to make a 172 amino acid length sequence for comparison to the allergen database. The bioinformatic results show that Ara h2 was clearly identified (Table 1); similarity with Ara h1 and Ara h3 was not identified as there were only four total proteins aligned and all of them were Ara h 2 isoforms. The additional hypothetical sequence containing a contiguous 66 amino acids section of the Ara h 2 protein (Fig. 1B) was more effective in identifying Ara h 1 and Ara h 3 (Supporting Information Table 2). The only other peanut allergen identified by this additional comparison and studied by Bublin et al. was Ara h 6. A primary reason a larger portion of the Ara h 2 protein was required to identify the nonhomologous, but cross‐reactive proteins, is the likely presence of undiscovered epitopes that are shared among these proteins. In contrast, the disparate epitope locations across the three proteins appear to have limited the ability to clearly identify shared similarity that may exist for this unique example of nonhomologous cross‐reactivity. For example, the epitope regions in Ara h1 and Ara h3 are located far away from the N‐terminal location 20. The Ara h1 region begins at amino acid position 584, and Ara h 3 begins at amino acid 328, whereas Ara h 2 begins very near the N‐terminal end at position 26 for the peptide AH2‐1.

## 4 Discussion

The premise behind comparing novel protein sequences to allergens has always been based on either identifying a known allergen or identifying a risk of allergic cross‐reactivity. Presumably, unexpected cross‐reactivity with a novel protein, or newly discovered protein, could happen in one of three ways. First, a portion of a known allergen could be inadvertently used to construct a synthetic novel protein. Second, a novel protein is derived from a species not yet identified as an allergen source and the novel protein is unexpectedly similar to its homologous counterpart, a known allergen. The third, and most unlikely scenario, involves the unintentional modification of a protein that results in enough similarity to share IgE binding with an allergen. For all practical purposes, even heavily modified novel proteins have to retain their function and structure to the extent that they are highly similar to the native protein expressed in the source organism. This level of retained, native similarity to a known structural class of protein makes it unlikely that random modification would somehow create an unexpected, but immunologically relevant level of similarity with an allergen. Otherwise, it is straightforward to identify the taxonomic class of the source organism and the structural family and function of the modified novel protein.

Well‐characterized allergen epitopes were used to examine the sensitivity of bioinformatics as a screening tool. The goal was to examine the level of sensitivity for detecting known cross‐reactivity potential by focusing on epitope sequence isolated from the rest of the parental core sequence. The B‐cell epitope was considered the minimum level of biologically relevant sequence that could identify the parental allergen or homologues for which cross‐reactivity risk could be observed.

The results demonstrated that FASTA retained its usefulness in identifying localized areas of sequence similarity even when the localized areas are small. This is an extension of the concept discussed by Bannon and Ogawa 4 and experimentally tested using maize allergen sequences 27, as well as with motif‐centric experiments where shared sequence across proteins is predicted first, then tested for immune reactivity 28. In this article, the Bet v 1 exercise was the best example of detecting homology at a very low proportion of native allergen sequence, with only 16 total residues representing a single discontinuous epitope (Fig. 1D). The 16 residues, although not contiguous within the hypothetical sequence, created enough of an exact match within the larger sequence to identify Bet v 1 (Table 1). This is because spacing along the length of the hypothetical sequence was the same as in endogenous Bet v 1 and thus, still allowed FASTA to identify this unique pattern of similarity; any other order would lower the similarity score. In this regard, the results hint at the importance of the underlying unique structure of the whole protein and the inherent spacing along the sequential sequence for identifying similarity between sequences with FASTA.

The summary statistic, the *E*‐value, performed well in identifying both the parental sequence from which the epitope was derived as well as homologous proteins with known cross‐reactivity. This was improved upon (lower *E*‐value and more homologous proteins from related species) when the whole Bet v 1 region was loaded into the random sequence, or when there were many more residues as there are for Ara h 1 and tropomyosin. In the case of tropomyosin, the epitopes are well conserved and unique to a degree that the *E*‐value range remained virtually unchanged even when the epitopes were inserted into a much longer (700 amino acids) hypothetical sequence (data not shown). In contrast to only lengthening the random amino acid content, a reduction from six to four total epitopes (two replaced with random sequence) was used as a separate comparison. This shifted the *E*‐value range in an increasing direction; the lowest value moving from 3.4 × 10^−27^ to 2.1 × 10^−15^, and the highest moving from 1.5 × 10^−09^ to 1 × 10^−06^, plus one additional alignment at *E*‐value = 7.4. The reduction in epitopes was impactful, but with such a highly conserved protein there was no reduction in the ability to detect homologues.

**Figure 2:**
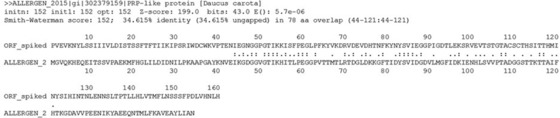
Alignment of the Bet v 1 epitope region (loaded into random amino acid sequence) with a PRP protein from carrot (*Daucus carota*).

The sensitivity in detecting similarity was much better using an *E*‐value rather than other metrics such as percent identity, or a combination percent identity and overlap length (Table 2). For example, *E*‐value was consistent in grouping known homologous Bet v 1 sequences. This is in contrast to the inconsistent designation by percent identity and overlap. Those alignments with *E*‐values below 10^−1^ and also having <35% identity and an overlap length <80 are shaded in gray in Table 2. An *E*‐value of 10^−1^ was considered as a minimum to survey for the accuracy of all alignments with regard to the percentage and overlap metrics. Clearly, members of the Bet v 1 family were identified by >35% identity and 80 or more amino acid overlap (Codex metrics), but these metrics were comparatively poor at identifying all of the homologous proteins. In the most extreme example, where only the Bet v 1 epitope residues were loaded into the hypothetical sequence, no alignments exceeding Codex metrics were observed below an *E*‐value of 10. Interestingly, the threshold in *E*‐value observed with proteins from carrot (*Daucus carotus*) is in line with the threshold of 3.9 × 10^−7^ calculated by Silvanovich et al. 27, but ranges just past this cutoff; *E*‐values 5.3 × 10^−5^ to 5.7 × 10^−6^. These values are very close to one another considering that the *E*‐value is typically judged on a log scale and both would be considered statistically significant 29. However, the hypothetical Bet v 1 protein and *Daucus* alignment displays percent identity and overlap length values just below thresholds of 35 and 80, respectively; an example in which those metrics 12 do not identify an important alignment from a screening perspective (Fig. 2). This would be important to recognize with regard to cross‐reactivity given the recent confirmation of IgE reactivity in Bet v 1 sensitized patients to the Dau c 1 protein 30. A lack of vicilin (vicilin‐like) homogues to the Ara h 1 hypothetical sequence was noted. A further evaluation noted the fact that some expected homologues, such as those from *Lupinus* and *Pisum*, were not present until the display limit was lowered to 25% identity. Yet, these same *Lupinus* and *Pisum* allergens had *E*‐values ranging from ∼1 × 10^−10^ to 1 × 10^−16^, further indicating that percent identity can be disconnected from the more relevant overall similarity identified by *E*‐value.

The more simplistic Codex metrics also fail to discriminate the tropomyosin and Ara h 2 region of epitopes (Supporting Information Tables 1 and 2). Alignments with the Pen a 1 hypothetical protein, for example, all align with significant *E*‐values, but the percent identity values decline to point where percent identity and overlap length do not coincide with obvious homology among the tropomyosins. This is also highlighted by the Bet v 1 analysis where the recently confirmed cross‐reactivity with Vig r 6 and Vig r 1 31 from *Vigna radiata* was noted by identification of these using an *E*‐value, but would not have been noted using Codex metrics (Table 2). The impact of lower percentages of epitope residues, relative to the rest of the allergen, is highlighted even more clearly by the inability to identify homologues for the Bet v 1 epitope. Compared with using *E*‐values, there were no alignments in the first 14 best results identified where either 35% identity or 80 amino acid lengths were observed (Table 3).

An *E*‐value of 1 × 10^−5^–1 × 10^−6^ has been identified that delineates significant allergen similarity, as modeled for the Ara h 1, Pen a 1, and Bet v 1 (region) epitopes. This *E*‐value range was selected based on the observation that *E*‐values lower than 1 × 10^−5^ were exclusively observed for the Pen a 1 and Ara h 1 hypothetical sequences (Table 1). In addition, the Bet v 1 epitope region‐containing hypothetical sequence displayed a breakpoint between cross‐reactive and other, nonhomologous sequences near this *E*‐value (Table 2). When modeling shorter proteins (the Bet v 1 protein is only 160–161 amino acids) with very limited epitope sequence in a discontinuous structure, a higher threshold may be appropriate (*E*‐value = 1 × 10^−3^) to identity similarity. There is some minimum level of sequence information that is below the point at which FASTA can consistently produce the same threshold *E*‐value compared with alignments based on the analysis of their full length sequence. The combination of a short protein and a single, dispersed epitope accounts for this impact.

Modeling of nonhomologous Ara h 2 cross‐reactive proteins 20 requires further clinical confirmation and further bioinformatic modeling in order to identify a meaningful *E*‐value threshold. In addition, *E*‐values based solely on Bet v 1 and a single discontinuous cross‐reactive epitope seem too indistinct to be considered predictive at this time. It is likely that the epitopes for both Bet v1 and the Ara h 1/Ara h 2/Ara h 3 complex are so uniquely distributed they may produce bioinformatic outcomes distinct from modeling observations of other allergens. Thus, identifying a single bioinformatic threshold for similarity using FASTA *E*‐values would be unlikely to hold true for various other discontinuous epitopes or nonhomologues proteins, respectively. This points to limitations in trying to fit a single threshold value across the many disparate groups of allergens. In the case of nonhomologues proteins, the epitopes may have arisen from cross‐reactivity due to very subtly distinct secondary and tertiary protein structures that simply cannot be resolved in the experiment herein where the epitopes have been taken away from contextual core sequence that would otherwise help identify homologous regions of proteins (i.e., more numerous local aligning sequences).

In combination with a well‐curated allergen database, allergen sequence screening benefits from an observed reduction in false‐positive alignments when using an *E*‐value of 1 × 10^−5^–1×10^−6^. More important from a safety perspective, false negatives based on percent identity were avoided by using an *E*‐value. A range, rather than a single value, is appropriate due to the unique nature of individual proteins and cross‐reactive allergen groups, which promotes thresholds that are expected to vary to some degree. Certainly, for full length proteins, an *E*‐value of 1 × 10^−5^ would be an effective *E*‐value threshold. Nevertheless, the overall conclusion is consistent with the basic tenant of allergen cross‐reactivity that there is no known risk of cross‐reactivity without homology to a known allergen. FASTA has the capability to serve this screening purpose given the appropriate application of the algorithm. The use of alignment parameters based on modeling of known allergens (both epitopes and core structure) that incorporate a relevant level of statistical significance criteria (i.e., *E*‐value) is the key.

Taken together, epitopes do appear to weight a random amino acid string enough to identify significant similarity and potential cross‐reactivity. In reality, allergens do not consist of just epitopes; they have core sequence structure that gives them their unique secondary and tertiary structure. Core sequence homology among related organisms is a key to the FASTA calculation of probable similarity because it was designed to help identify conserved domains 10. In terms of using a dedicated allergen database, shared core sequence is the basis for relatedness among allergens 32, 33. When at least some of the core sequence is present (e.g. Bet v 1 epitope region) or when epitopes are relatively numerous, FASTA easily identified homologues and produced an *E*‐value threshold that was indicative of statistically significant alignments according to FASTA expectations. The bioinformatic screening of sequences based on FASTA *E*‐values would be consistent with the intent of the FASTA algorithm statistical metrics for homology and an improvement over shared identity and overlap length. Examinations into the details of all of the alignments within an allergen class are always advised once screening for homology identification is performed.

The study herein complements previous work 27, 34, 35, 36 intended to identify biologically relevant metrics for screening novel proteins. The first attempts at creating regulatory thresholds for novel protein screening were based on percent identity and sequence overlap length, and they have persisted until present day 12, 13. These values were primarily based on Bet v 1 homology structure, but it was unclear how these would perform using curated allergen databases such as FARRP 16, which were not available at the time. There are more sophisticated informatics methods that have been adapted for specific antigens 37. And, for deep analyses it is unlikely that one method is able to completely capture the variability across all known homologues of a given structural class 38, much less all of those different classes in an allergen database. Yet, a local‐alignment approach based on shared similarity and the use of an accepted general standard is a way forward since it underpins the very use of algorithms such as FASTA and the similar BLAST. In effect, by using the summary *E*‐value statistic, the base percent identity and length of alignment is incorporated into an analysis of similarity between proteins. As presented, the concept of building an analysis of cross‐reactivity among known allergens offers a “from the ground up” approach that can be extended. It has the potential to support modeling of the different allergen structural groups to identify meaningful thresholds for shared similarity, as it remains to be identified whether there is a single shared feature of proteins that make them allergens. The goal is to support the growing knowledge base of understanding the nature of how allergens are similar in structure, function, and their propensity to cross‐react in order to work toward a predictive approach that is both conservative and accurate.

No conflict of interest. Employed solely by agricultural biotechnology company, Syngenta Crop Protection, LLC.

## Supporting information

Supplemental Figure 1. The alpha‐amylase gene from human (NCBI Genbank identification 565263) was used as the source for the alternative frame open reading frame sequences used as filler sequence to prepare each of the hypothetical sequences.

Supplementary Tables
