# Supplementary material for: Bioinformatic screening and detection of allergen cross‐reactive IgE‐binding epitopes
Source: Mol Nutr Food Res. 2017 Mar 27;61(8):1600676. doi: 10.1002/mnfr.201600676 (PMC5573986; doi:10.1002/mnfr.201600676)
Supplement: Supplementary file 1 — Supplemental Figure 1. The alpha‐amylase gene from human (NCBI Genbank identification 565263) was used as the source for the alternative frame open reading frame sequences used as filler sequence to prepare each of the hypothetical sequences. [file MNFR-61-na-s001.docx]

Supplemental Figure 1.

Open Reading Frame 1: human_amylase_gi_565263_gb_AH002671.1_121 [1337 - 1029] (REVERSE SENSE)

PVEVKNYLSSIIIVLDISTSSFTFTIIKIPSRIWDCWKVPTSRVEVTSTGTACSCTHSITTHMINYSIHINTNLENNSLTPTLLHLVTMFLNSSSFPDLVHNL

Open Reading Frame 2: human_amylase_gi_565263_gb_AH002671.1_91 [2342 - 1935] (REVERSE SENSE)

HHSMCQQEDQFAKLKKMSCKIIYLPVIIVENNESSVSSPKSHLVAPIIIPVCKRLAIHYIAEINHVPKNTYSHLIWRHRCSQTQSLPQVVSGLIVTSLVTPLSFGGPTQSLTSLITQNLRFHFQNILANGSLTLLV
