# Supplementary material for: Bioinformatic screening and detection of allergen cross‐reactive IgE‐binding epitopes
Source: Mol Nutr Food Res. 2017 Mar 27;61(8):1600676. doi: 10.1002/mnfr.201600676 (PMC5573986; doi:10.1002/mnfr.201600676)
Supplement: Supplementary file 2 — Supplementary Tables [file MNFR-61-na-s002.docx]

Supplementary Table 1. All alignments comparing the hypothetical protein containing the Pen a 1 Tropomyosin epitopes with the allergen database. Rows indicated by shaded cells in the E-value column have a % identity < 35 and/or an overlap < 80.

| **Database Match Description** | **GI #** | **Species** | **%Identity** | **Overlap** | **E-Value** |
| --- | --- | --- | --- | --- | --- |
| Tropomyosin | 607633 | *Metapenaeus ensis* | 43.9 | 244 | 3.4E-27 |
| Fast Tropomyosin Isoform | 2660868 | *Homarus americanus* | 44.9 | 245 | 3.5E-27 |
| Tropomyosin | 288819271 | *Macrobrachium rosenbergii* | 43.9 | 244 | 3.5E-27 |
| Lit V 1 Tropomyosin | 170791252 | *Litopenaeus vannamei* | 43.9 | 244 | 4.1E-27 |
| Pen A 1 Allergen | 73532979 | *Farfantepenaeus aztecus* | 43.9 | 244 | 4.1E-27 |
| Tropomyosin | 60892782 | *Penaeus monodon* | 43.9 | 244 | 4.1E-27 |
| Tropomyosin Fast Isoform | 125995159 | *Marsupenaeus japonicus* | 43.9 | 244 | 4.1E-27 |
| Tropomyosin | 162286975 | *Oratosquilla oratoria* | 43.5 | 248 | 4.9E-27 |
| Tropomysin | 225348412 | *Procambarus clarkii* | 43.9 | 244 | 5.8E-27 |
| Fast Muscle Tropomyosin | 3080761 | *Panulirus stimpsoni* | 44.5 | 245 | 7.8E-27 |
| Tropomyosin Fast Isoform | 125995163 | *Paralithodes camtschaticus* | 44.5 | 245 | 9.4E-27 |
| Tropomyosin | 448278534 | *Portunus pelagicus* | 44.1 | 245 | 2.1E-26 |
| Tropomyosin | 148615631 | *Tyrophagus putrescentiae* | 43 | 244 | 4.1E-26 |
| Tropomyosin | 238477263 | *Crangon crangon* | 43 | 244 | 4.9E-26 |
| Tropomyosin | 558698675 | *Macrobrachium rosenbergii* | 43 | 244 | 5.8E-26 |
| Tropomyosin | 156712754 | *Euphausia pacifica* | 41.5 | 248 | 9.4E-26 |
| Tropomyosin, Allergen Pan B 1 | 312831088 | *Pandalus borealis* | 42.2 | 244 | 2.5E-25 |
| Tropomyosin | 156712752 | *Euphausia superba* | 40.7 | 248 | 3.5E-25 |
| Allergen Tropomyosin | 119674937 | *Portunus sanguinolentus* | 41.4 | 244 | 4.9E-25 |
| Tropomyosin | 151505279 | *Scylla serrata* | 41.4 | 244 | 4.9E-25 |
| Tropomyosin | 151505281 | *Portunus trituberculatus* | 41.4 | 244 | 4.9E-25 |
| Tropomyosin | 134305330 | *Eriocheir sinensis* | 41.4 | 244 | 4.9E-25 |
| Tropomyosin Slow-Twitch Isoform | 125995169 | *Erimacrus isenbeckii* | 41.4 | 244 | 5.8E-25 |
| Slow Tropomyosin Isoform | 2660866 | *Homarus americanus* | 42.3 | 241 | 3E-24 |
| Tropomyosin Slow-Tonic Isoform | 125995165 | *Paralithodes camtschaticus* | 40.8 | 240 | 4.9E-24 |
| Tropomyosin Slow-Tonic Isoform | 125995171 | *Erimacrus isenbeckii* | 39.8 | 244 | 8E-24 |
| Tropomyosin | 48249227 | *Tyrophagus putrescentiae* | 38.9 | 244 | 2.1E-23 |
| Mag44 | 1359436 | *Dermatophagoides farinae* | 39.1 | 243 | 2.6E-23 |
| Tropomyosin | 208970286 | *Dermatophagoides pteronyssinus* | 39.5 | 248 | 3E-23 |
| Group 10 Allergen Blo T 10 | 156938889 | *Blomia tropicalis* | 38.7 | 243 | 3.5E-23 |
| Tropomyosin | 2353266 | *Dermatophagoides pteronyssinus* | 39.1 | 248 | 3.5E-23 |
| Tropomyosin; Slow-Tonic Isoform; Tm-Chio | 308191588 | *Chionoecetes opilio* | 40.6 | 244 | 4.9E-23 |
| Tropomyosin | 2440053 | *Dermatophagoides pteronyssinus* | 38.7 | 248 | 1.5E-22 |
| Lep D 10 Protein | 6900304 | *Lepidoglyphus destructor* | 38.3 | 243 | 2.5E-22 |
| Tropomyosin | 80553470 | *Dermatophagoides pteronyssinus* | 38.3 | 248 | 5.7E-22 |
| Tropomyosin | 4468639 | *Periplaneta americana* | 38.4 | 242 | 9.4E-22 |
| Tropomyosin | 4378573 | *Periplaneta americana* | 38 | 242 | 2.1E-21 |
| Tropomyosin | 8101069 | *Blattella germanica* | 38 | 242 | 2.1E-21 |
| Tropomyosin | 19310971 | *Periplaneta fuliginosa* | 36.9 | 241 | 4.1E-21 |
| Tropomyosin | 239740599 | *Periplaneta americana* | 38 | 242 | 6.8E-21 |
| Tropomyosin | 83715932 | *Todarodes pacificus* | 36.2 | 246 | 1.5E-20 |
| Tropomyosin | 83715928 | *Sepia esculenta* | 36.2 | 246 | 2.5E-20 |
| Tropomyosin | 83715930 | *Sepioteuthis lessoniana* | 35.8 | 246 | 4.9E-20 |
| Tropomyosin | 83715934 | *Ommastrephes bartramii* | 35.8 | 246 | 4.9E-20 |
| Tropomyosin | 4468224 | *Helix aspersa* | 33.3 | 264 | 4.9E-20 |
| Tropomyosin | 7321108 | *Chironomus kiiensis* | 37.3 | 241 | 5.8E-20 |
| Tropomyosin | 83715936 | *Octopus vulgaris* | 34.9 | 241 | 4.9E-19 |
| Tropomyosin | 224016002 | *Ascaris lumbricoides* | 34.7 | 248 | 5.8E-19 |
| Tropomyosin | 14423976 | *Anisakis simplex* | 35.7 | 249 | 6.8E-19 |
| Tropomyosin | 9954249 | *Haliotis diversicolor* | 33.2 | 244 | 6.8E-19 |
| Tropomyosin | 219806586 | *Haliotis discus discus* | 33.2 | 244 | 8E-19 |
| Tropomyosin | 219806573 | *Venerupis philippinarum* | 33.6 | 247 | 8E-19 |
| Tropomyosin | 350285785 | *Anisakis simplex* | 35.3 | 249 | 1.8E-18 |
| Tropomyosin | 125659386 | *Balanus rostratus* | 33.5 | 251 | 2.5E-18 |
| Tropomyosin | 219806596 | *Fulvia mutica* | 33.6 | 247 | 3.5E-18 |
| Tropomyosin; Altname: Mov-14; Ov-Tmy-1 | 42559586 | *Onchocerca volvulus* | 33.1 | 248 | 4.9E-18 |
| Tropomyosin | 20387027 | *Lepisma saccharina* | 33.7 | 246 | 4.9E-18 |
| Tropomyosin | 219806600 | *Tresus keenae* | 33.9 | 251 | 8.0E-18 |
| Tropomyosin | 15419048 | *Crassostrea gigas* | 35 | 200 | 8.3E-18 |
| Heat Stable Allergen Tropomyosin | 7024506 | *Charybdis feriatus* | 37 | 227 | 1.1E-17 |
| Tropomyosin | 219806588 | *Turbo cornutus* | 34.1 | 252 | 1.3E-17 |
| Tropomyosin | 219806594 | *Crassostrea gigas* | 32.4 | 241 | 1.5E-17 |
| Tropomyosin | 219806590 | *Neptunea polycostata* | 32.4 | 241 | 2.1E-17 |
| Tropomyosin | 219806598 | *Pseudocardium sachalinensis* | 33.1 | 251 | 2.1E-17 |
| Tropomyosin | 219806602 | *Solen strictus* | 32.4 | 250 | 4.1E-17 |
| Tropomyosin | 219806592 | *Scapharca broughtonii* | 32.5 | 240 | 4.9E-17 |
| Tropomysin | 156145810 | *Sinonovacula constricta* | 32.1 | 252 | 6.8E-17 |
| Tropomyosin | 9954253 | *Chlamys nobilis* | 31.4 | 245 | 1.3E-15 |
| Tropomyosin | 9954251 | *Perna viridis* | 31.5 | 200 | 4.1E-14 |
| Troposmyosin | 20387029 | *Lepisma saccharina* | 38.5 | 161 | 1E-13 |
| Tropomyosin | 3668408 | *Crassostrea virginica* | 35.2 | 159 | 2.4E-13 |
| Tropomyosin | 156938915 | *Tyrophagus putrescentiae* | 35.7 | 129 | 1.5E-09 |

Supplementary Table 2. All alignments comparing the hypothetical protein containing the Ara h 2 overlapping peptides with the allergen database. A 66 amino acid region was inserted spanning the epitopes AH2-1, AH2-2 and AH2-3a AH2-3b and AH2-3c. Rows indicated by shaded cells in the E-value column have a % identity < 35 and/or an overlap < 80.

| **Database Match Description** | **GI #** | **Species** | **%Identity** | **Overlap** | **E-Value** |
| --- | --- | --- | --- | --- | --- |
| allergen Ara h 2 isoform | 31322017 | *Arachis hypogaea* | 74 | 96 | 1.20E-25 |
| allergen Ara h 2.02 | 26245447 | *Arachis hypogaea* | 74 | 96 | 1.20E-25 |
| Ara h 2 | 15418705 | *Arachis hypogaea* | 64.8 | 88 | 1.80E-18 |
| Ara h 2.01 allergen | 224747150 | *Arachis hypogaea* | 64.8 | 88 | 1.90E-18 |
| Conglutin; AltName: Ara h 6 | 75114094 | *Arachis hypogaea* | 33.8 | 77 | 4.00E-04 |
| Chain A, Allergen Arah6 From Peanu | 159163254 | *Arachis hypogaea* | 48.9 | 47 | 4.10E-04 |
| allergen Arah6 | 5923742 | *Arachis hypogaea* | 32.5 | 77 | 1.30E-03 |
| Allergen Bra j 1-E (Bra j I) | 32363444 | *Brassica juncea* | 31.7 | 41 | 8.40E-01 |
| A5A4B3 subunit | 806556 | *Glycine soja* | 30.6 | 62 | 2.30E+00 |
| glycinin | 3703107 | *Arachis hypogaea* | 31.6 | 79 | 2.40E+00 |
| arachin Arah3 isoform | 199732457 | *Arachis hypogaea* | 31.6 | 79 | 2.90E+00 |
| glycinin | 5712199 | *Arachis hypogaea* | 31.6 | 79 | 4.40E+00 |
